# Gross morphological and ultrasonographic dimensions of normal feline kidney with reference to resistive index

**DOI:** 10.1038/s41598-024-75341-0

**Published:** 2024-10-27

**Authors:** Mohamed Ahmed Maher, Laura Palacio, Juan C. Henao, Samar H. Elsharkawy

**Affiliations:** 1 Department of Anatomy and Embryology, Faculty of Veterinary Medicine, Cairo University, Giza Square, P.O. 12211, Cairo, Egypt; 2 Department of Small Animal Medicine and Surgery, Xavier University School of Veterinary Medicine Aruba “XUSOVM”, Dutch Caribbean, Oranjestad, Aruba; 3 Department of Surgery, Anesthesiology and Radiology, Faculty of Veterinary Medicine, Cairo University, Giza Square, P.O. 12211, Cairo, Egypt

**Keywords:** Doppler ultrasonography, Resistive index, Inter-lobar artery, Anatomy, Cat, Nephrology, Urology

## Abstract

Ultrasonography with color Doppler is the most quantitative analysis method for intra-renal parameters. There is a wide variation between authors in the measurement site and referencing the inter-lobar resistivity index (RI) in felines. Our objective is to morphometrically and ultrasonographically investigate the normal renal dimensions and vasculatures and draw up a normal reference value for the renal inter-lobar artery resistivity index (RI) using a Pulsed wave-Doppler ultrasonography. A total of twelve adult domestic cats were sedated and treated according to IACUC regulation guidelines to be examined using Doppler ultrasound. The same cats were used for morphometric investigation and divided into three groups regarding the used technique. The size difference between the right and left kidneys is slight, measuring 17 g (weight), 3.65 ± 0.06 cm (length), 2.54 ± 0.08 cm (width), and 2.21 ± 0.03 cm (thickness) for the right kidney, and about 15 g, 3.42 ± 0.06 cm, 2.32 ± 0.05 cm, and 2.13 ± 0.03 cm for the left one, respectively. There are three patterns of renal arteries’ point of origin. The mean RI values of both kidneys were 0.57 ± 0.08 (0.50–0.67) in the right kidney and 0.60 ± 0.08 (0.51–0.69) in the left kidney. The gross examination and ultrasonography measurements did not have a statistically different effect on the actual renal dimensions. Moreover, 0.69 is considered the standard resistivity index (RI) threshold of the feline inter-lobar artery with no correlation to the animal’s body weight.

## Introduction

The kidney is a fundamental organ for maintaining water and electrolyte equilibrate in individuals and various animal species. The second most important function of this organ is to eliminate multiple harmful metabolic waste products, specifically nitrogenous substances like urea and creatinine^1^. In both urology and nephrology practice, renal parameters are highly useful diagnostic indicators for both individuals and animals^2^. Due to their close structural mimicry of the human kidney, pig kidneys are more commonly used in urologic experimental studies^3^ while cats are the most appropriate models for bilateral renal ischemia^4^.

The kidneys are vascularized through the renal vasculatures. The left renal artery is shorter and originates after the right one^5^. Before entering the opposite hilus, each artery is divided into dorsal and ventral divisions in dogs^6,7^, in cats^2^, in rats^8^, and in goats^9^. In the cat, there are 4 branches for each dorsal and ventral branch, while in the dog, there are 2 branches for each. The primary branch is made up of the dorsal and ventral divisions, which are then subdivided into inter-lobar, arcuate, and interlobular arteries, respectively^2,5,9,10^. In certain canine kidneys, the renal hilus receives two or more renal arteries^11^ and from one to six primary branches^12^.

A decrease in renal arterial diastolic flow is usually indicative of increased renal arterial resistance, which is linked to acute renal dysfunctions like tubular necrosis, impaired perfusion, and obstructions. Practically, the resistivity index (RI) is frequently ascertained by Doppler ultrasonography because it is the most affordable diagnostic protocol. The renal arterial system’s inter-lobar and arcuate expressions at the hilus or cortico-medullary zone are the source of the signal-dependent nature of this technique. During systolic and diastolic blood flow, the prevalence shift is utilized to give a proportional measurement of velocity because small arteries are not accessible. Normally, the resistive index referencing in cats does not exceed 0.70. So, the elevated index indicates an elevated resilience and subsequently, a distinct renal disease such as renal arterial stenosis^13–17^.

This investigation intends to scrutinize the morphometric anatomical parameters of the feline kidneys and related vasculatures in adult domestic cats in Egypt and pinpoint the variations between other studied mammals. Moreover, to set a referenced metric for the feline renal dimensions and inter-lobar arteries RI for each kidney, using B-mode and pulsed wave-Doppler ultrasonography. Finally, comparing dimensions driven from ultrasonography to actual measurements obtained from gross morphology.

## Methods

### Ultrasonography

Our study included 12 adult healthy cats of different sexes (6 males and 6 females) and weights (2.65∼5.3 kg) based on physical, ultrasonographic and laboratory investigations. The ultrasonographic survey was conducted by placing the cats in a dorsal position after IM administration of xylazine (1 mg/kg). The ventral abdomen was lavishly coated with a hydro-soluble gel to permit sound conduction. The sonographic scanning procedure was executed with a stationary articulated arm (Mindray DC-40, Shenzhen, China) with a micro-convex (5-9 MHz) transducer. All kidneys were assessed in B-Mode through multiple axial scans made at 0.5-cm intervals, determining its size, shape, echotexture, cortex: medulla ratio and evaluation of renal pelvis and ureter. Renal parameters including width, length, height and thickness (cortical and medullary) were obtained at three planes (dorsal, transverse and sagittal planes)^18,19^. The renal length and height (cm) were determined from the sagittal plane (Fig. 8) at the frame where echogenic parallel segments formed by pelvic diverticulum were clearly spotted. The renal width (cm) and height (cm) were measured from the transverse plane (Fig. 9), where the “C”-sign of the renal crest (Fig. 9/RC) was visualized^18^. On the dorsal plane (Fig. 10) where the kidney in a true bean shape with the renal sinus in the far field, both renal length and width were recorded. At the sagittal plane, the cortical thickness (cm) was determined as the distance connecting the renal margin to the corticomedullary junction, while the medullary thickness (cm) was determined as the interface between the corticomedullary junction to the renal pelvis^19^.

Renal inter-lobar arteries were appointed using color flow-Doppler then pulsed wave- Doppler tracings were obtained by positioning a gate of 2.5 mm wide over the artery, setting the wall filter to 125 Hz, and choosing the smallest scale. Finally, the peak systolic (PSV) and end-diastolic (EDV) velocities were averaged from three consecutive waveforms, and RI was calculated automatically using the following formula^18^.

$${\text{RI }} = \left( {{\text{PSV }}{-}{\text{ EDV}}} \right)/{\text{PSV}}.$$

Furthermore, all renal parameters were compared with actual parameters obtained from gross morphology.

### Anatomical studies

The present investigation was conducted on the same twelve adult domestic cats of both sexes that appeared healthy by physical and ultrasonographic examination. All the cats were sedated with xylazine (1 mg/kg), and anesthesia was induced using ketamine (20 mg/kg) followed by Heparin (5000 IU/ml) injected intramuscularly to prevent coagulation. To overcome the pain, an overdose of sodium thiopental 2.5% was used then exsanguination following the rules established by the institutional animal care and use committee. The institutional animal care and use committee (IACUC) of Cairo University has approved the present work with an IACUC number of (VET CU 03162023730). All methods were performed in accordance with the relevant guidelines and regulations and were reported in accordance with ARRIVE guidelines 2.0. A cannula through the common carotid artery was fixed to clear the remaining blood within the vessels using a phosphate-buffered saline of 0.9% followed by a buffered formalin solution of 10%. For studying the renal angioarchitecture, the animals were divided into 3 groups according to the used technique with 4 cats for each. The radiographic group; using a contrast medium as Omnipaque™, injected through the abdominal aorta just prior to the cranial mesenteric artery then immediately radiographed using 55 KVP 30–70 MA, 0.5 s and FFD70 cm. The latex-injected group; using red paint milky latex injected as the radiographic group and then immersed in 10% formalin solution for fixation. The corrosion-cast group; using KEMAPOXY 150 (2 A/1B) was mixed in a ratio of 2 A:1B then injected immediately by manual push using syringes of 50 ml before solidification in a room temperature for 48 h then maceration of the cast was applied by using 40% KOH for 5 days. After that, the specimens were dissected and cleaned carefully.

The specimens were anatomically dissected in situ then removed from the sub-lumbar region to be measured for weight and related dimensions before maceration using KOH solution then photographed using a digital photo camera Nikon Coolpix L310 14.1 Megapixels. The nomenclature used in this study was adopted according to Nomina Anatomica Veterinaria (2017), as well as those used by previous literature on carnivores.

### Statistical analysis

Pulsed wave- Doppler measurements for both right and left inter-lobar arteries were statistically analyzed with IBM SPSS statistics version 26 to determine intragroup variability. The comprehensive data were presented as (mean ± SD) and all P values lesser than 0.05 were considered significantly different.

## Results

### Morphometry of feline kidneys

**Fig. 1:**
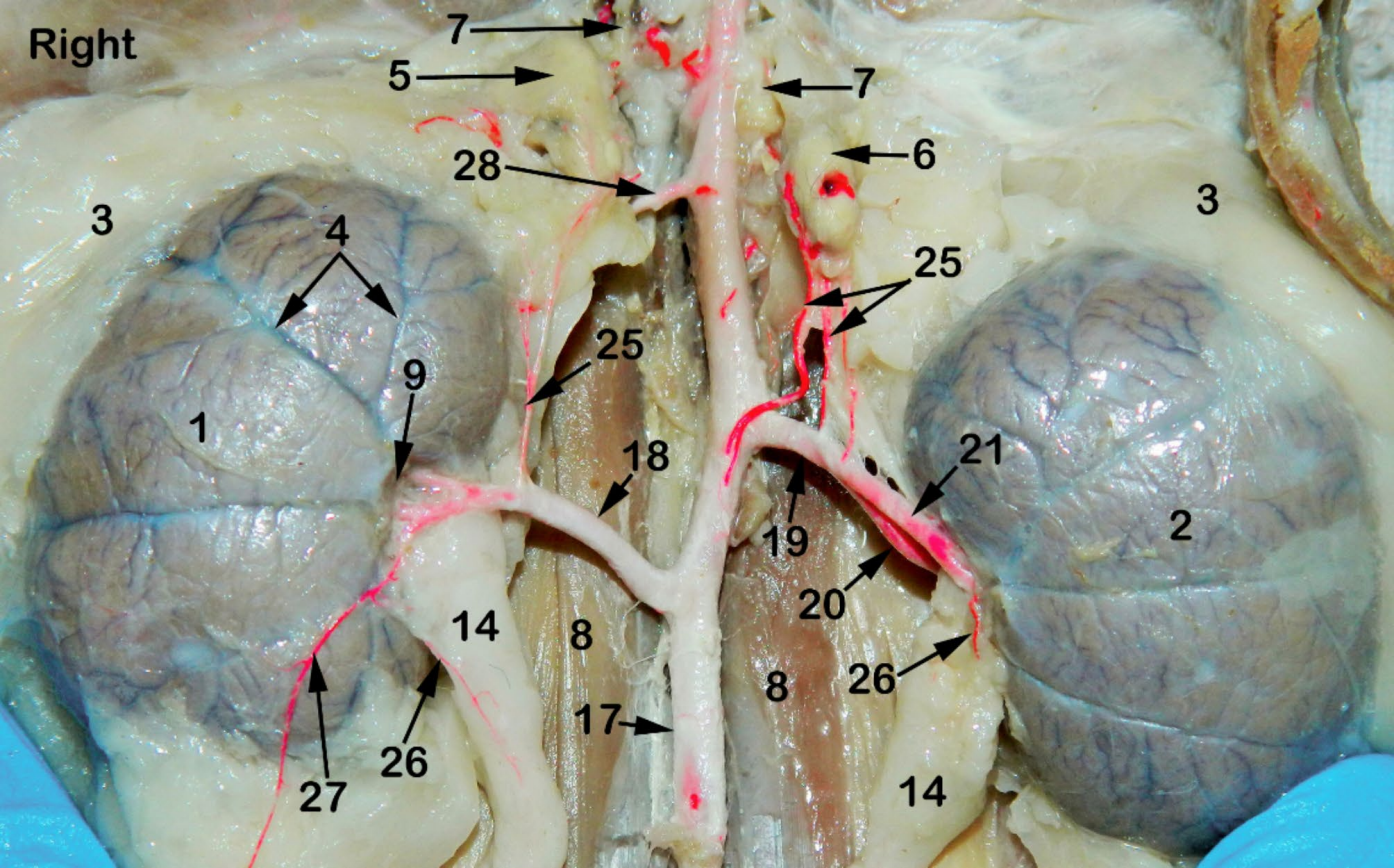
A photograph showing ventral view of abdominal cavity of cat showing kidney position, abdominal aorta and renal vasculature. 1, right kidney; 2, left kidney; 3, peri-renal fat; 4, sub-capsular veins; 5, right adrenal gland; 6, left adrenal gland; 7, renal lymph nodes; 8, sublumbar muscles; 9, renal hilus; 10, renal pelvis; 11, cortico-medullary zone; 12, Cortex; 13, renal capsule; 14, ureter; 15, 1st lumbar vertebra; 16, 3rd lumbar vertebra; 17, Abdominal aorta; 18, right renal artery; 19, left renal artery; 20, dorsal primary branch; 21, Ventral primary branch; 22, inter-lobar artery; 23, arcuate artery; 24, Inter-lobular artery; 25, caudal adrenal arteries; 26, ureteric branch; 27, capsular branch; 28, lumbar arteries.

**Fig. 2:**
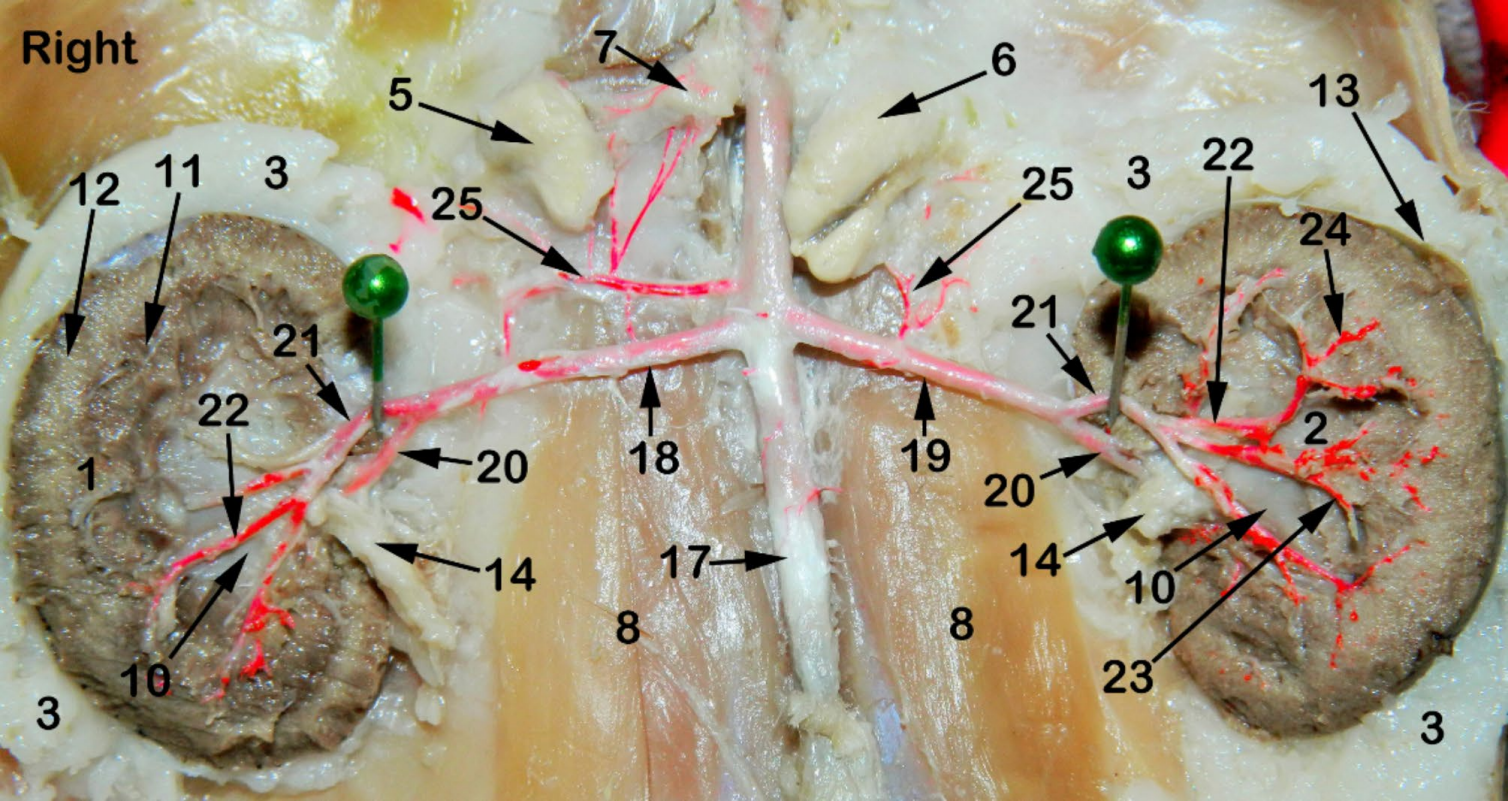
A photograph showing ventral view of abdominal cavity of cat showing kidney position, abdominal aorta and renal artery ramification through the kidneys. 1, right kidney; 2, left kidney; 3, peri-renal fat; 4, sub-capsular veins; 5, right adrenal gland; 6, left adrenal gland; 7, renal lymph nodes; 8, sublumbar muscles; 9, renal hilus; 10, renal pelvis; 11, cortico-medullary zone; 12, Cortex; 13, renal capsule; 14, ureter; 15, 1st lumbar vertebra; 16, 3rd lumbar vertebra; 17, Abdominal aorta; 18, right renal artery; 19, left renal artery; 20, dorsal primary branch; 21, Ventral primary branch; 22, inter-lobar artery; 23, arcuate artery; 24, Inter-lobular artery; 25, caudal adrenal arteries; 26, ureteric branch; 27, capsular branch; 28, lumbar arteries.

**Fig. 3:**
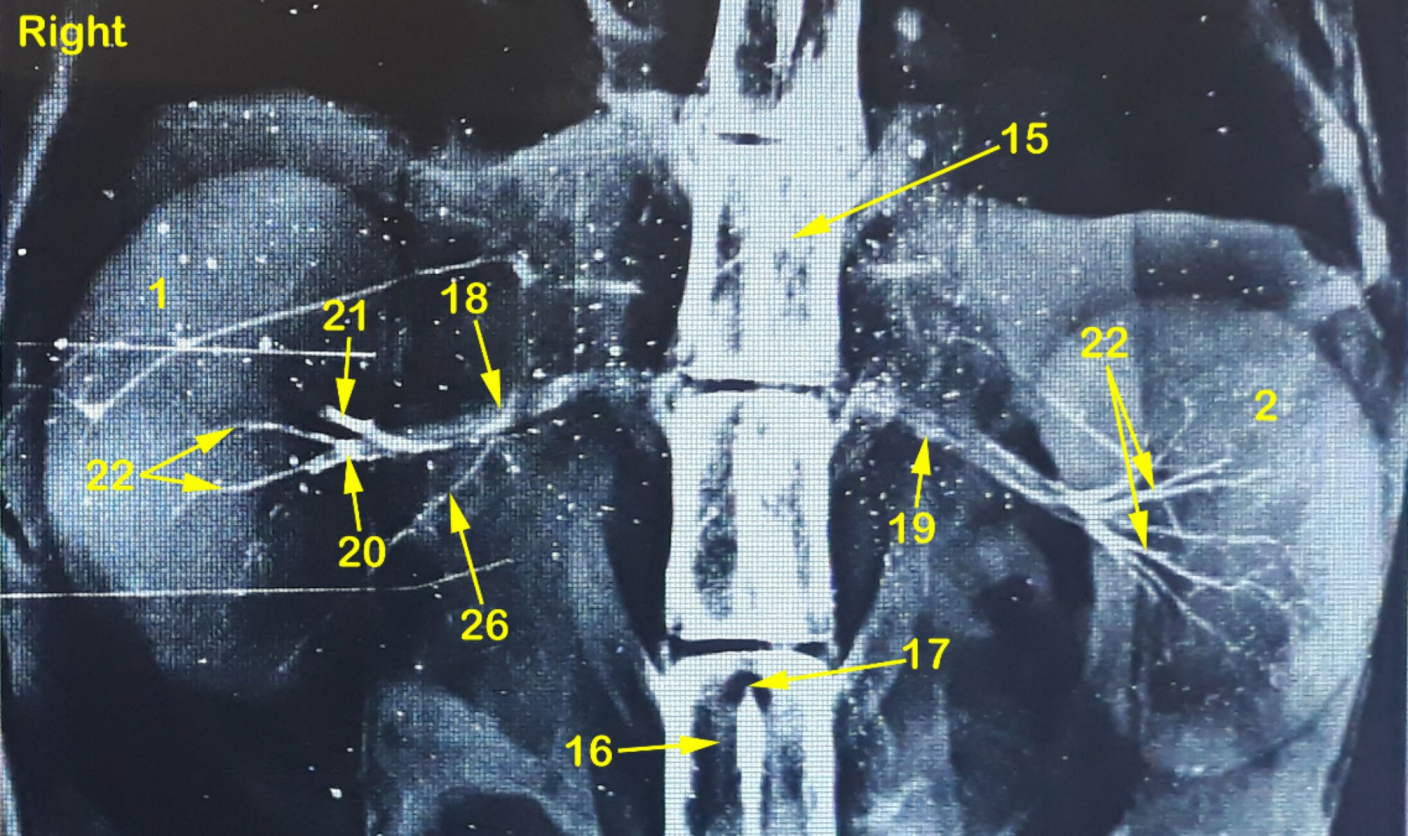
A radiograph showing dorso-ventral view of abdominal cavity of cat showing kidney position, renal artery point of origin and its ramification through the kidneys. 1, right kidney; 2, left kidney; 3, peri-renal fat; 4, sub-capsular veins; 5, right adrenal gland; 6, left adrenal gland; 7, renal lymph nodes; 8, sublumbar muscles; 9, renal hilus; 10, renal pelvis; 11, cortico-medullary zone; 12, Cortex; 13, renal capsule; 14, ureter; 15, 1st lumbar vertebra; 16, 3rd lumbar vertebra; 17, Abdominal aorta; 18, right renal artery; 19, left renal artery; 20, dorsal primary branch; 21, Ventral primary branch; 22, inter-lobar artery; 23, arcuate artery; 24, Inter-lobular artery; 25, caudal adrenal arteries; 26, ureteric branch; 27, capsular branch; 28, lumbar arteries.

**Fig. 4:**
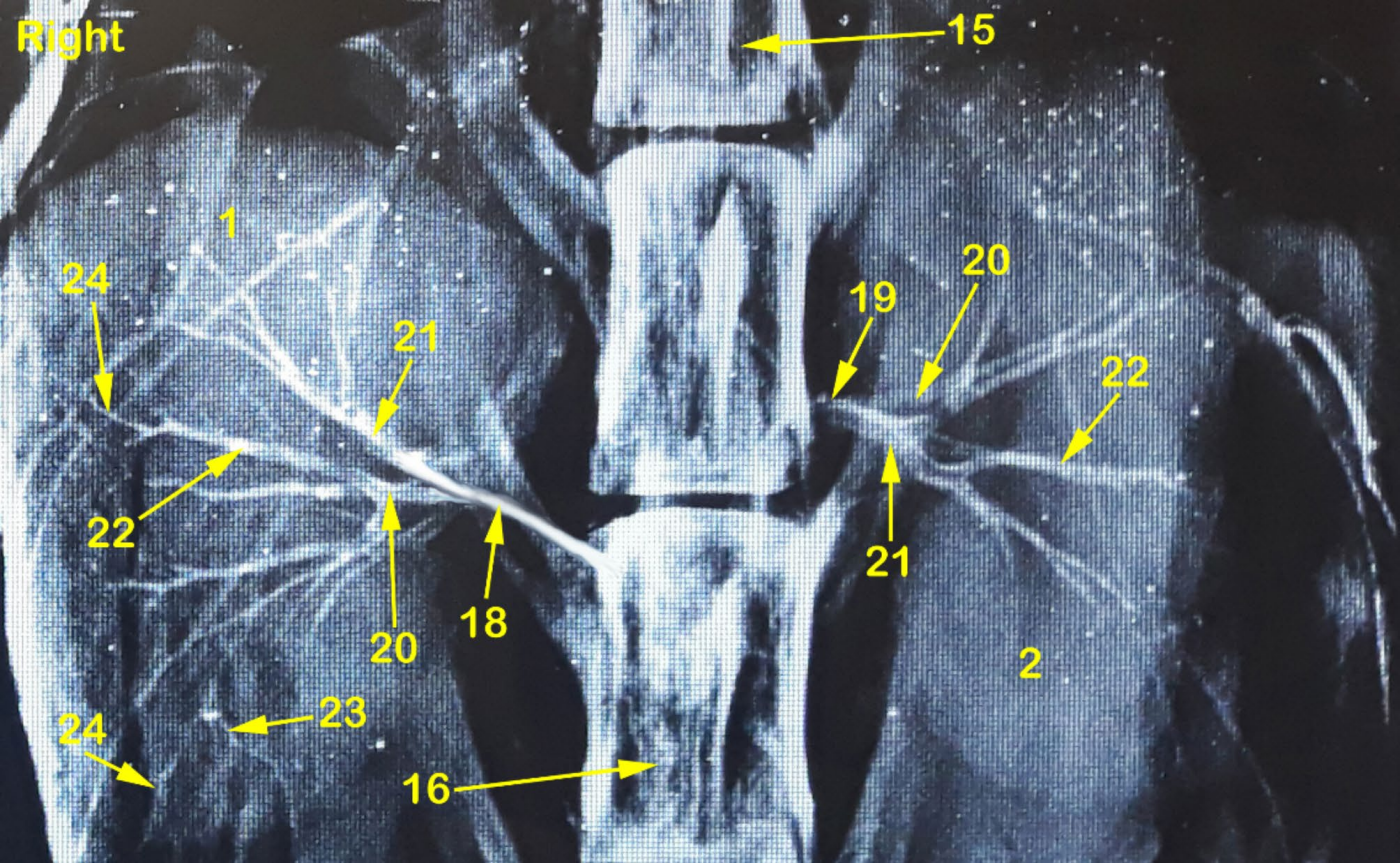
A radiograph showing dorso-ventral view of abdominal cavity of cat showing kidney position, renal artery point of origin and its ramification through the kidneys. 1, right kidney; 2, left kidney; 3, peri-renal fat; 4, sub-capsular veins; 5, right adrenal gland; 6, left adrenal gland; 7, renal lymph nodes; 8, sublumbar muscles; 9, renal hilus; 10, renal pelvis; 11, cortico-medullary zone; 12, Cortex; 13, renal capsule; 14, ureter; 15, 1st lumbar vertebra; 16, 3rd lumbar vertebra; 17, Abdominal aorta; 18, right renal artery; 19, left renal artery; 20, dorsal primary branch; 21, Ventral primary branch; 22, inter-lobar artery; 23, arcuate artery; 24, Inter-lobular artery; 25, caudal adrenal arteries; 26, ureteric branch; 27, capsular branch; 28, lumbar arteries.

**Fig. 5:**
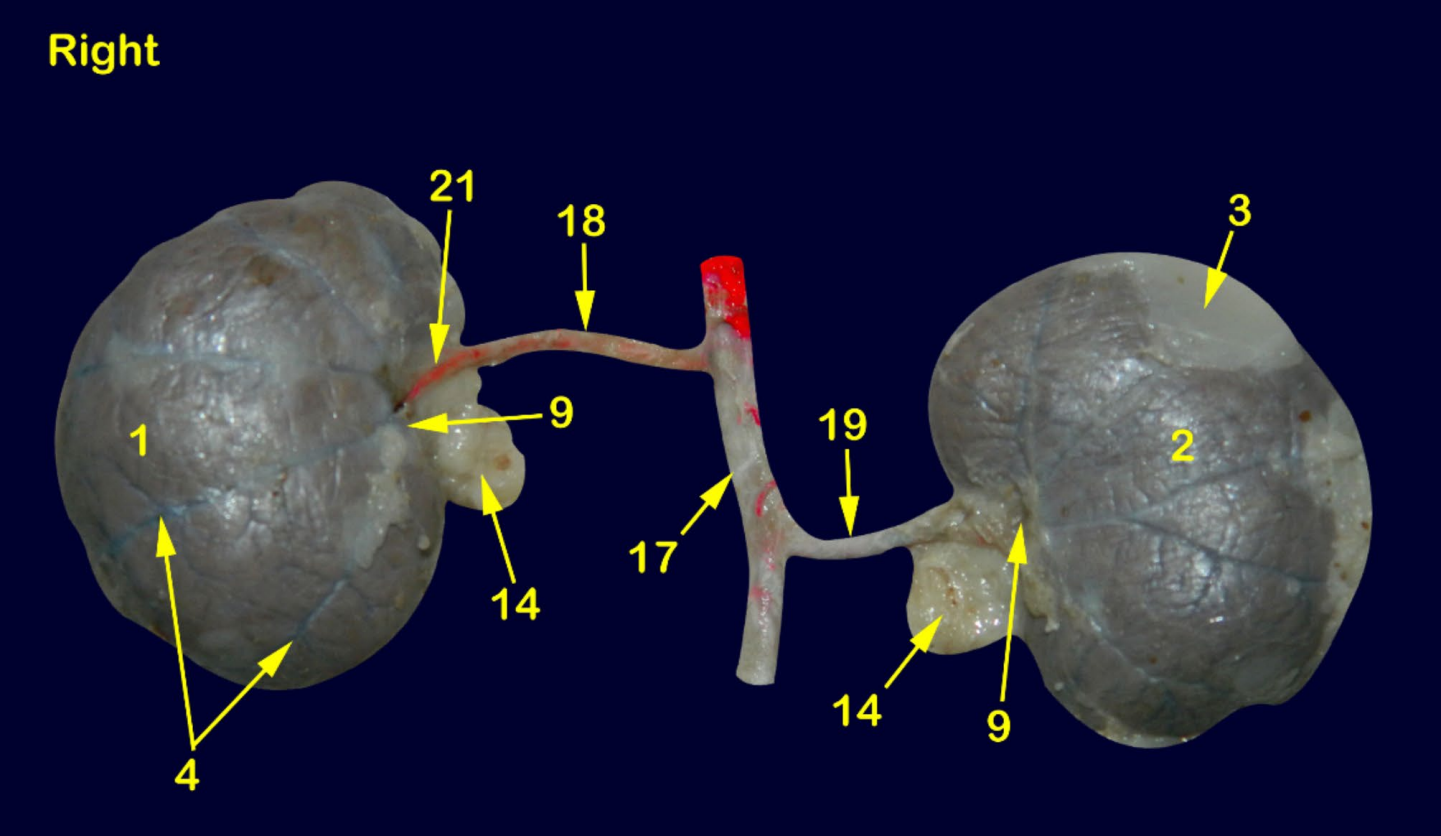
A photograph showing ventral view of cat kidneys injected with red-colored latex. 1, right kidney; 2, left kidney; 3, peri-renal fat; 4, sub-capsular veins; 5, right adrenal gland; 6, left adrenal gland; 7, renal lymph nodes; 8, sublumbar muscles; 9, renal hilus; 10, renal pelvis; 11, cortico-medullary zone; 12, Cortex; 13, renal capsule; 14, ureter; 15, 1st lumbar vertebra; 16, 3rd lumbar vertebra; 17, Abdominal aorta; 18, right renal artery; 19, left renal artery; 20, dorsal primary branch; 21, Ventral primary branch; 22, inter-lobar artery; 23, arcuate artery; 24, Inter-lobular artery; 25, caudal adrenal arteries; 26, ureteric branch; 27, capsular branch; 28, lumbar arteries.

The feline kidney appeared bean in outline with a smooth surface externally and asymmetrically localized in the sub-lumbar region both kidneys are situated under the first three lumbar transverse processes (Figs. 1, 2, 3 and 4) with rounded cranial and caudal poles, dorsal and ventral aspects, lateral convex and medial hilar concave boundaries (Fig. 5). The hilar border is depressed at its middle receiving arterial supply, nerve plexus and emitting venous drainage, ureter, and lymphatics. Both kidneys are covered by thick fatty capsules and thin tight fibrous ones that reveal the characteristic bright-blue feather-like appearance of the sub-capsular veins (Figs. 1 and 5).

### Morphometry of renal arterial supply

**Fig. 6:**
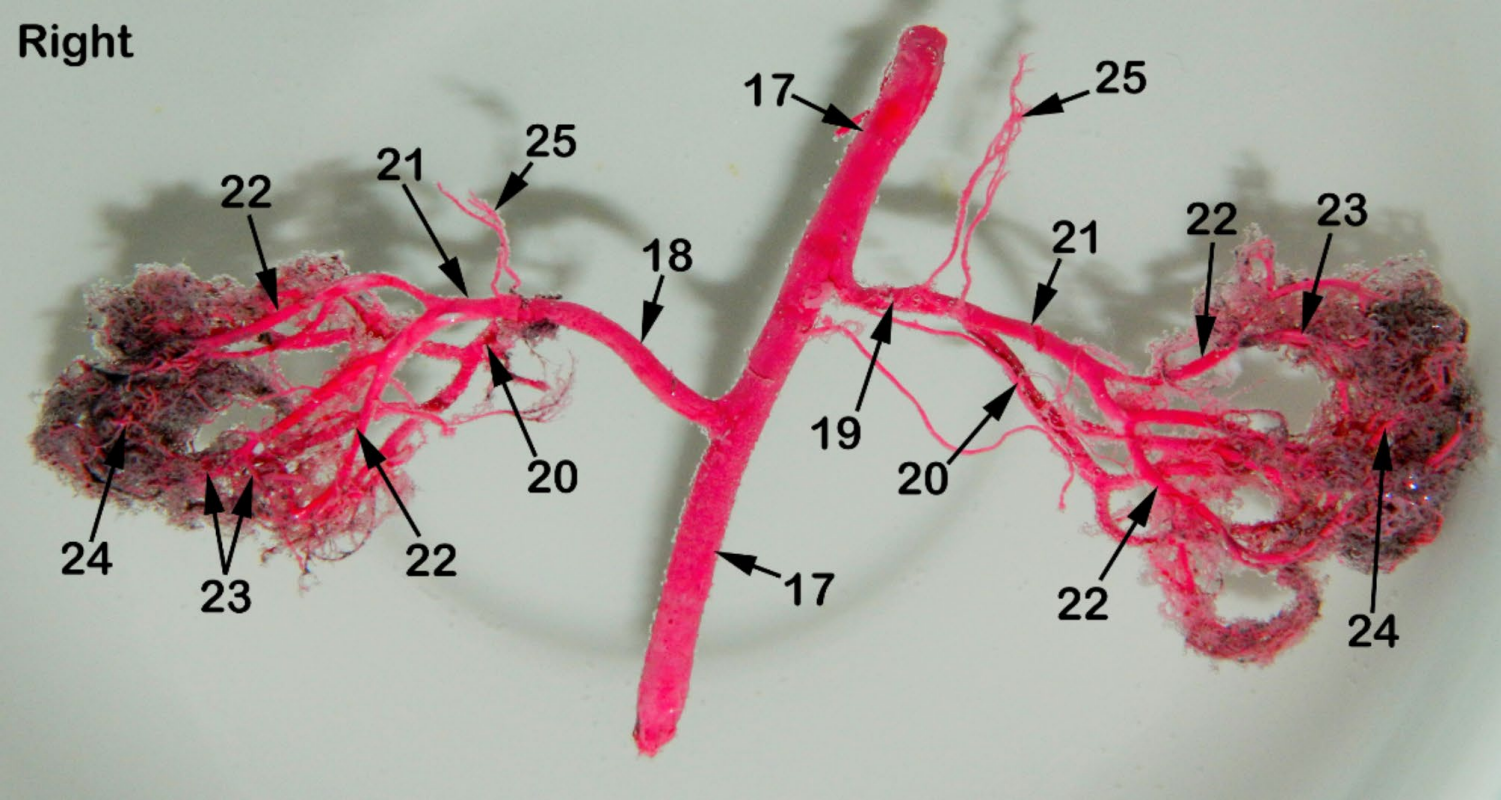
A photograph showing a cast of red-colored latex revealing the distribution of renal arteries through the renal parenchyma of cat. 1, right kidney; 2, left kidney; 3, peri-renal fat; 4, sub-capsular veins; 5, right adrenal gland; 6, left adrenal gland; 7, renal lymph nodes; 8, sublumbar muscles; 9, renal hilus; 10, renal pelvis; 11, cortico-medullary zone; 12, Cortex; 13, renal capsule; 14, ureter; 15, 1st lumbar vertebra; 16, 3rd lumbar vertebra; 17, Abdominal aorta; 18, right renal artery; 19, left renal artery; 20, dorsal primary branch; 21, Ventral primary branch; 22, inter-lobar artery; 23, arcuate artery; 24, Inter-lobular artery; 25, caudal adrenal arteries; 26, ureteric branch; 27, capsular branch; 28, lumbar arteries.

**Fig. 7:**
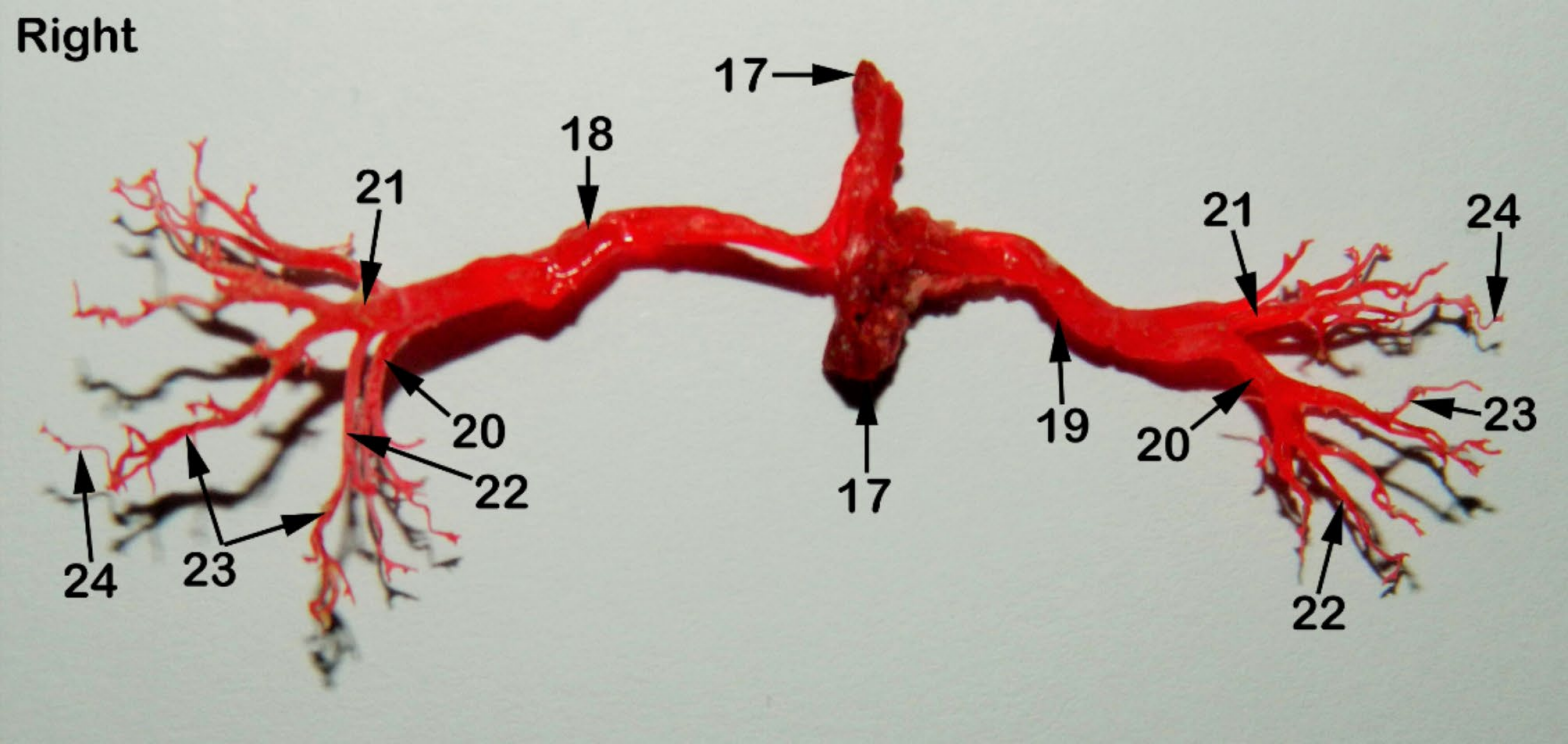
A photograph showing a corrosion cast of red-colored epoxy revealing the distribution of renal arteries through the renal parenchyma of cat. 1, right kidney; 2, left kidney; 3, peri-renal fat; 4, sub-capsular veins; 5, right adrenal gland; 6, left adrenal gland; 7, renal lymph nodes; 8, sublumbar muscles; 9, renal hilus; 10, renal pelvis; 11, cortico-medullary zone; 12, Cortex; 13, renal capsule; 14, ureter; 15, 1st lumbar vertebra; 16, 3rd lumbar vertebra; 17, Abdominal aorta; 18, right renal artery; 19, left renal artery; 20, dorsal primary branch; 21, Ventral primary branch; 22, inter-lobar artery; 23, arcuate artery; 24, Inter-lobular artery; 25, caudal adrenal arteries; 26, ureteric branch; 27, capsular branch; 28, lumbar arteries.

The Aa. Renales dexter et sinister originate from the sideward margin of the aorta abdominalis, measuring about 0.33 ± 0.02 cm in diameter, midway between the 2nd and 3rd lumbar vertebral bodies (Figs. 2 and 3). The renal arteries’ point of origin revealed main three patterns of distribution. In the 1st pattern, there are 7 cats out of 12 with a percentage of 58.3%, where the A. renalis sinister originated earlier than the A. renalis dexter (Figs. 1 and 6). In the 2nd pattern, there are 3 cats out of 12 with a percentage of 25%, where the Aa. Renales dexter et sinister have emerged at the same level opposite to each other (Figs. 2, 3 and 7). In the 3rd pattern, the A. renalis dexter originated earlier than the sinister one in 2 cats with a percentage of 16.6% (Fig. 5). Both renal arteries are directed laterally toward their renal hilus after dividing into two primary divisions; dorsal (Fig. 6/20) and ventral branches (Fig. 6/21).

There is no accessory renal artery has been observed in all studied cats. The renal artery length from its root to the primary splitting up is 1.79 ± 0.35 cm and 1.28 ± 0.47 cm for the right and left renal arteries, respectively. The diameter of the right and left renal arteries is 0.17 ± 0.03 cm and 0.15 ± 0.02 cm, respectively. The adrenal glands (Fig. 2/5, 6) and renal lymph nodes (Fig. 1/7) receive fine arterial branches that originated in 8 cats from the corresponding renal artery (Fig. 1/25) while in 4 cats, the left gland is irrigated by the left renal artery, and the right gland from a separate branch originated from the abdominal aorta (Fig. 2/25). The dorsal and ventral primary divisions of each renal artery are divided into cranial and caudal branches that are further divided into about 3–5 inter-lobar arteries for each (Fig. 4/22). The dorsal and ventral inter-lobar arteries are radiated from the renal hilus, enclosing the renal pelvis at its dorsal and ventral aspects, through the renal medulla till reaching the cortico-medullary zone where each inter-lobar artery is divided into two arcuate arteries (Fig. 6/23). The arcuate arteries gave rise to tiny interlobular arteries (Fig. 6/24) with a larger number than the arcuate arteries, distributed to the periphery of the renal parenchyma with no terminal anastomosis between them.

### Ultrasonographic evaluation

The renal dimensions, length, height, and width, of twelve domestic cats, were presented in (Table 1), as measured by different ultrasonographic angles with their resistive indices (RIs). The ultrasound-observed renal dimensions were identical to the actual dimensions measured by gross observations in (Table 2), with a non-significant difference (*p* > 0.05).

**Table 1:** Kidney dimensions and resistive index of 12 domestic cats measured in different ultrasonographic angles:

Renal dimensions		Sagittal plane	Transverse plane	Dorsal plane
Length	R	4.23 ± 0.84	N/A	3.86 ± 0.93
	L	3.90 ± 1.10	N/A	4.06 ± 0.95
Height	R	2.02 ± 0.62	2.07 ± 0.32	N/A
	L	2.12 ± 0.67	2.34 ± 0.51	N/A
Width	R	N/A	2.52 ± 0.29	2.23 ± 0.41
	L	N/A	2.73 ± 0.66	1.96 ± 0.49
Cortical thickness	R	0.53 ± 0.26	N/A	N/A
	L	0.55 ± 0.23	N/A	N/A
Medullary thickness	R	0.33 ± 0.11	N/A	N/A
	L	0.41 ± 0.18	N/A	N/A
RI	R	0.57 ± 0.08 (0.50–0.67)	N/A	N/A
	L	0.60 ± 0.08 (0.51–0.69)	N/A	N/A

**Table 2:** Actual sizes of renal dimensions of 12 domestic cats measured in necropsy using vernier calipers.

Renal dimensions		Actual size (Mean ± SD)
Renal artery length	R	1.79 ± 0.35
	L	1.28 ± 0.47
Renal artery diameter	R	0.17 ± 0.03
	L	0.15 ± 0.02
Kidney length	R	3.65 ± 0.06
	L	3.42 ± 0.06
Kidney thickness	R	2.21 ± 0.03
	L	2.13 ± 0.03
Kidney width	R	2.54 ± 0.08
	L	2.32 ± 0.05

Unit: cm; SD: standard deviation, L: left, R: right.

**Fig. 8:**
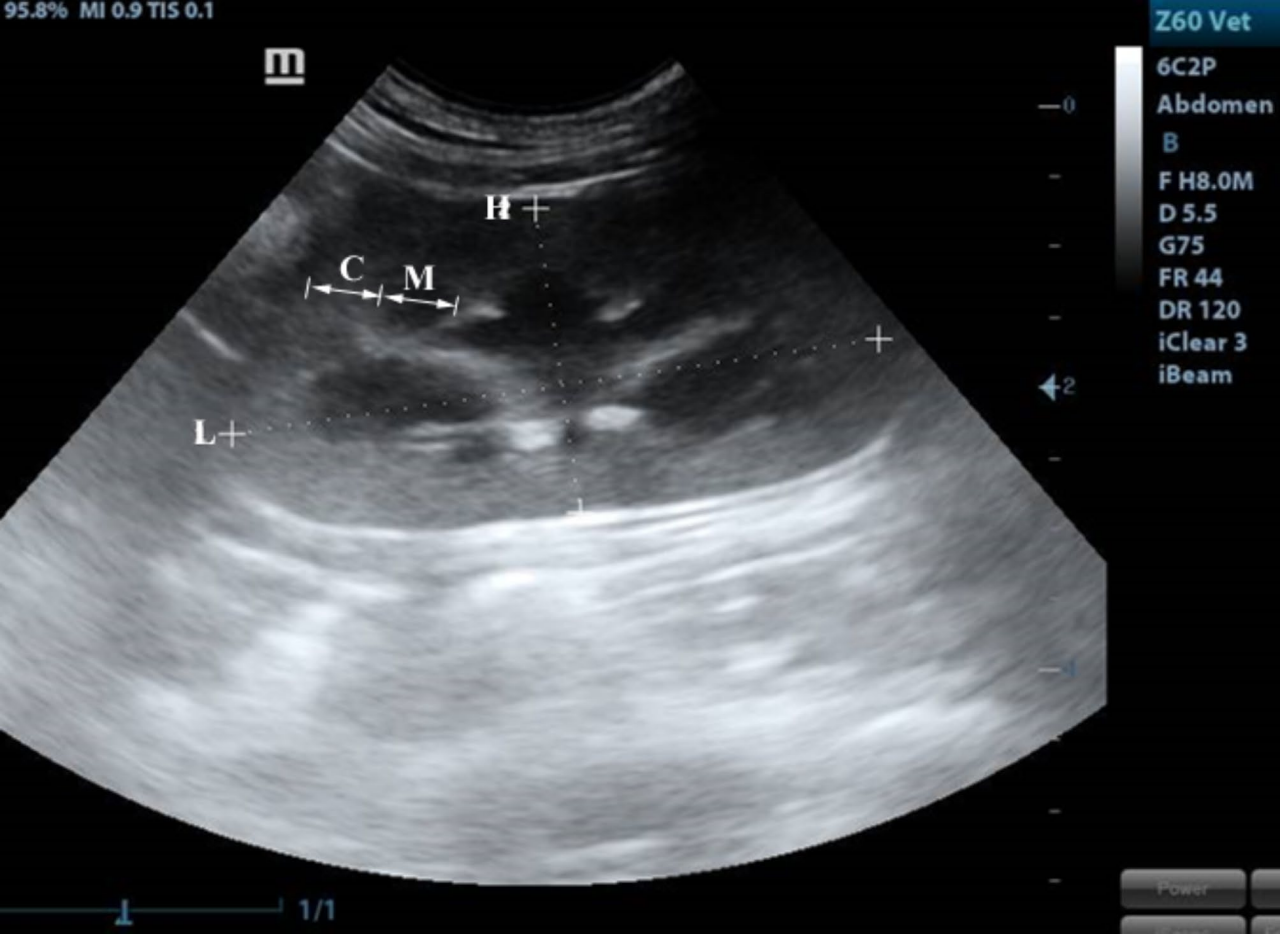
A sagittal plane of the kidney showing L: length, H: height, C: cortex, M: medulla.

**Fig. 9:**
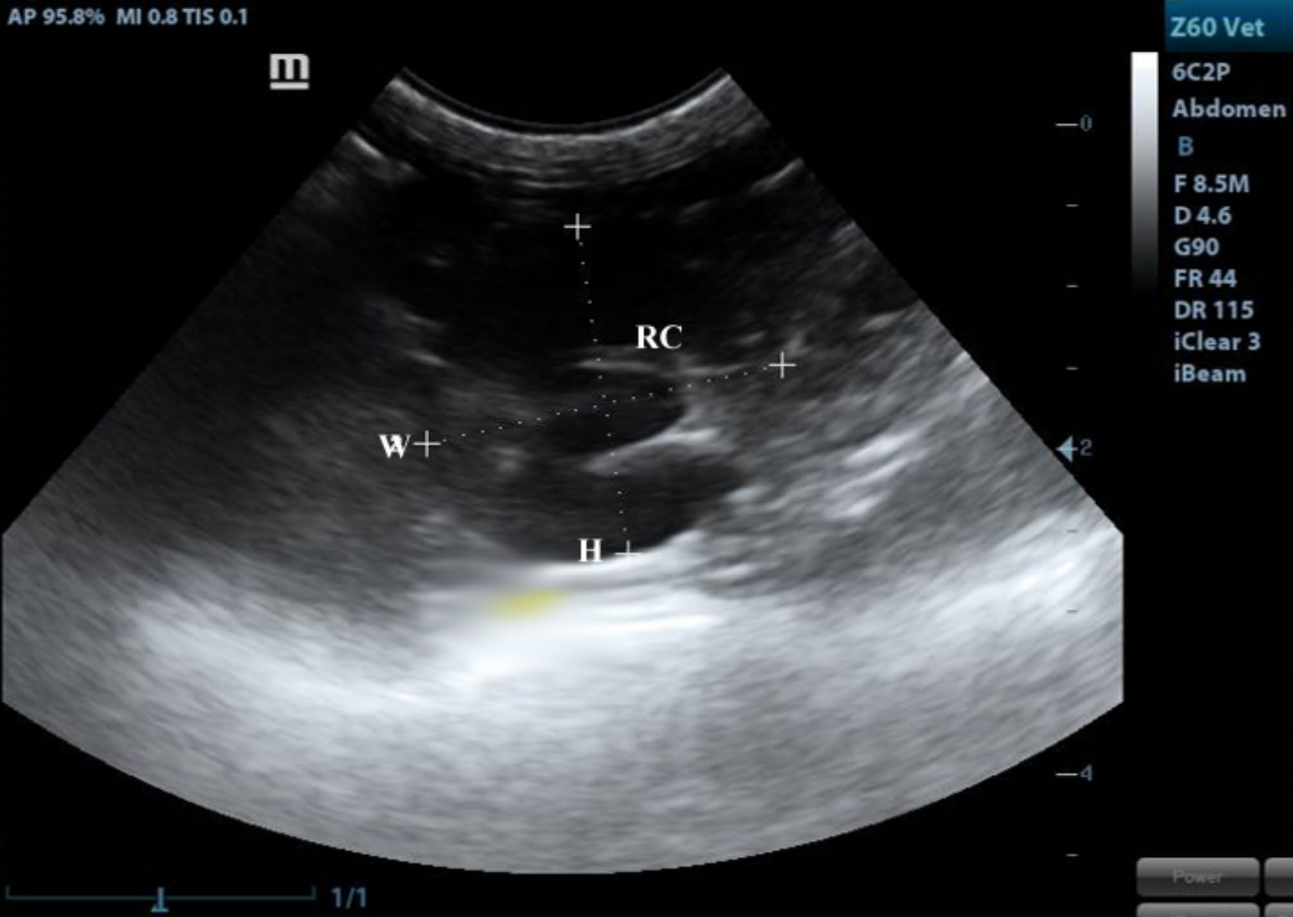
A transverse plane of the kidney showing renal crest (RC) appeared as “C”-sign. H: height, W: width.

**Fig. 10:**
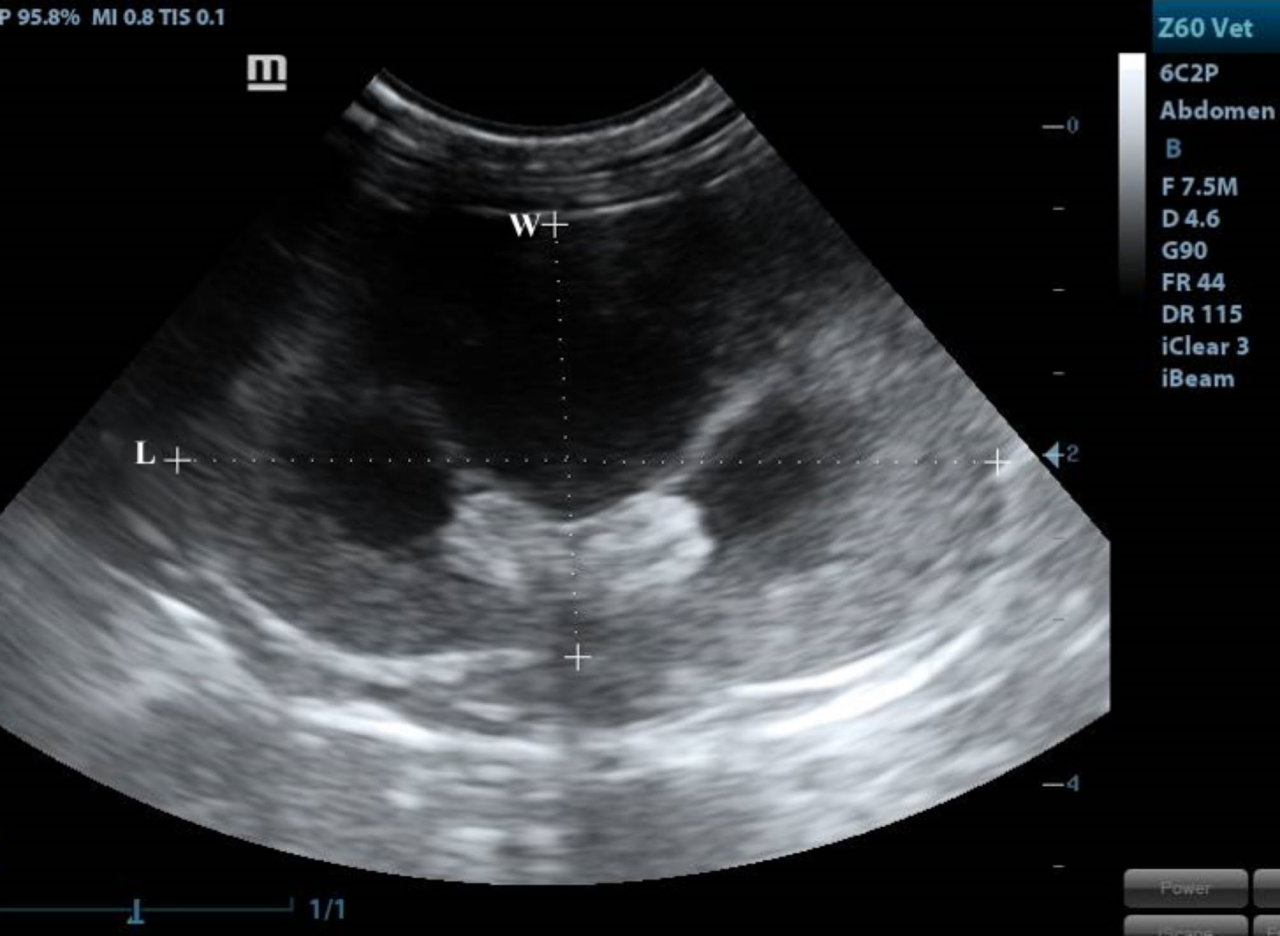
A dorsal plane of the kidney showing H: height, W: width.

Both kidneys showed normal sonographic appearance: smooth in texture, well-defined, oval, and had a thin, linear, hyperechoic renal capsule. The cortex of the right kidney was consistently echoic and somewhat isoechoic or hypoechoic when compared to the liver parenchyma, whereas, the cortex of the left kidney was consistently hypoechoic when compared to the splenic parenchyma, which appeared clear in the sagittal plane (Fig. 8). Linear echogenicities, such as recesses or diverticula, cross the medulla, forming a thin linear hyperechoic band, which is called the “C-sign” of the common renal crest in the transverse plane (Fig. 9). A series of dorsal scans were performed at intervals of 0.5 cm until the kidney’s most lateral border was obscured (Fig. 10).

**Fig. 11:**
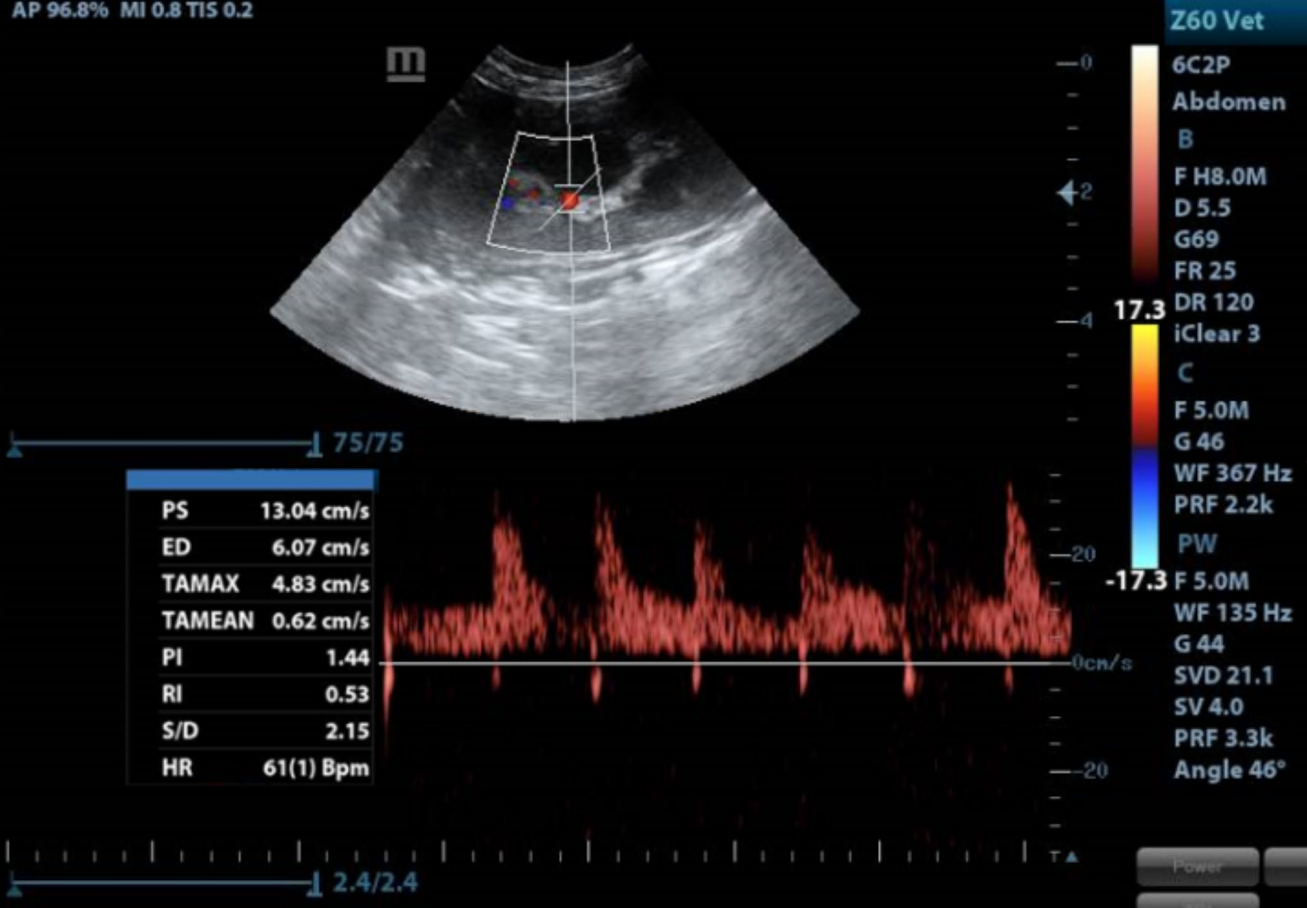
Determination of Resistivity index (RI) using color Doppler echocardiography.

The right kidney measures 17 g in weight, 3.65 ± 0.06 cm in length, 2.54 ± 0.08 cm in width, and 2.21 ± 0.03 cm in thickness. The left kidney measures 15 g, 3.42 ± 0.06 cm, 2.32 ± 0.05 cm, and 2.13 ± 0.03 cm, respectively. The mean thicknesses (mean ± SD) of the renal cortex and medulla were 0.53 ± 0.26 cm in the right kidney, 0.55 ± 0.23 cm in the left kidney, and 0.33 ± 0.11 cm in the right kidney and 0.41 ± 0.18 cm in the left kidney, respectively. The mean RI values of both kidneys were 0.57 ± 0.08 in the right kidney and 0.60 ± 0.08 in the left kidney (Fig. 11).

The cat populations included in this investigation ranged widely in body weight (2.65–5.3 kg), the correlation index was determined by statistically analyzing the measured kidney dimensions (Table 3). The body weight and renal medullary thickness had a higher degree of correlation with the renal dimensions, while the degree of correlation to the renal cortical thickness was low.

**Table 3:** Coefficient of correlation of body weight to renal dimensions.

Renal dimensions		Sagittal plane	Transverse plane	Dorsal plane
Length	R	0.662	N/A	0.702
	L	0.803	N/A	0.588
Height	R	0.736	0.429	N/A
	L	0.697	0.888*	N/A
Width	R	N/A	0.613	0.556
	L	N/A	0.807	0.901*
Cortical thickness	R	0.200	N/A	N/A
	L	0.535	N/A	N/A
Medullary thickness	R	0.746	N/A	N/A
	L	0.959**	N/A	N/A

Coefficient of correlation below 0.3, is considered as weak^33^. N/A; not applicable, L: left, R: right, * Correlation is significant at the 0.05 level (2-tailed), ** Correlation is significant at the 0.01 level (2-tailed).

## Discussion

The kidneys of our studied cats are smooth, bean-shaped, retroperitoneal, and asymmetrically, localized in the sub-lumbar region with three patterns of kidney positioning while previous literature^2,9,12,20,21^ had been reported that the right kidney is normally extending a little more cranial than the left one and covered by a thick fatty capsule. The current study revealed that the right kidney measurements were 3.65 ± 0.03 cm, 2.54 ± 0.04 cm, and 2.21 ± 0.01 cm in length, width, and thickness, respectively and the left kidney were 3.42 ± 0.03 cm, 2.32 ± 0.02 cm, and 2.13 ± 0.15 cm, respectively. Meanwhile, the cat’s right kidney measured about 3.54 ± 0.46 cm, 2.45 ± 0.27 cm, and 2.05 ± 0.23 cm, respectively and the left kidney were 3.52 ± 0.44 cm, 2.46 ± 0.28 cm, and 2.19 ± 0.31 cm, respectively^21^. The present study agreed with the previous reports that the right kidney was slightly heavier than the left kidney with the renal arteries originating from each side of the abdominal aorta^8,21,22^. On the other hand, in cats, confirmed that the left kidney was larger than the right one, especially in the male’s antimere^2^. In addition, in rabbits^23^, observed that the left kidneys were more affected by formalin fixation than the right kidneys in the form of decreased right kidney thickness.

Our observations revealed three patterns of renal arteries originating from the abdominal aorta. The 1st pattern, where the left renal artery emitted before the right one, the 2nd pattern, where the two arteries originated opposite to each other and the 3rd pattern, the right artery departed before the left one. In dogs^12^ reported the 3rd pattern in 69.23% and the 2nd pattern in 30.76% of studied dogs. However, in rabbits^24,25^ and in cats^20^ reported the 3rd pattern only with the right renal artery was longer than the left one, due to the position of the right kidney which was higher than the left one. Although the left renal artery was reported to be longer than the right one in cats^26^, some researchers found the opposite of this finding in some species of cats^27,28^. However, in all cases, our investigation was in agreement with^8^ that the right renal artery was slightly longer than the left one, due to the location of the aorta being left to the median plane of the abdominal cavity as humans. The present observations were in agreement with the previous reports in rabbits^29^, in goats^30^, in cats^26^, in pigs^31^, in wolves^32^ and in rats^8^ that there was a single renal artery in all examined kidneys. Otherwise, multiple renal arteries were examined and reported in humans^33^ and in dogs^12,22^.

Our results were similar to the previous reports that the primary division of each renal artery was a dorsal and ventral branches^8,9,26,32,34,]^. But others found that the primary division of the pig renal artery was a cranial and a caudal branch in 93.4% of cases^35^. While in humans^36^, recorded the primary divisions as anterior and posterior branches. Furthermore, in goats^9^, reported an accessory renal artery before the primary division. The results which not simulate the present study in cats. The current investigation revealed that the dorsal and ventral branches of each renal artery were divided into two segmental arteries, known as a cranial and a caudal branch which further divided to give off the inter-lobar arteries. These results were in agreement with^8,11^. On the contrary, other authors as in rabbits^29^, in goats^30^ in sheep^27^, and in wolves^11^, reported that the dorsal and ventral branches of renal arteries directly gave off the inter-lobar arteries.

Our observations in the present study were most relevant to those reported previously in cats^11,37^. The current renal dimensions obtained in the dorsal, sagittal and transverse planes were comparable to normal ranges for feline kidneys^18,35,39^. In the present study, Doppler hemodynamic changes were excluded by assessing the RI of the inter-lobar or arcuate arteries for each kidney. Our measurements were taken during sedation with an Alpha 2-adreneroceptor agonist, deemed reliable based on prior research showing no significant difference in renal PI and RI between awake and sedated cats using the same drug category^38^. RI for both kidneys showed no difference from the previous studies^18,35,40–44^. Normally, the resistive index differences between the right and left kidneys are not significant as reported in the previous studies^42–44^, however, a later study demonstrated that RI differed significantly between both kidney which was attributed to technical errors or even a clinically unappreciated kidney injury^45^. Our study population showed no difference in the RI between the right and left kidney as described before^35,42–44^.

There is a paucity of data on comparing actual renal dimensions with measurements obtained via ultrasound. Nonetheless, a study in 2008 demonstrated no difference between the two methods in domestic short hair cats which aligned with the findings of our study^35^. Renal measurements of the feline resistive index (RI) of the renal inter-lobar artery in the current investigation were nearly similar to those reported by^41^ in domestic short-hair cats where the RI values were 0.58 in the right kidney and 0.59 in the left kidney with no significant difference between different groups due to gender and right and left kidneys. Meanwhile, in cats^18^ reported the RI values as 0.75 ± 0.07 for the right kidney and 0.71 ± 0.05 for the left kidney.

## Conclusion

There is no difference between ultrasound driven renal measurements and actual renal measurements assessed by gross morphometrical analysis. RI obtained from renal inter-lobar arteries via pulsed wave Doppler is a body weight independent measurement in the feline population. In addition, we suggest 0.69 as an upper limit of a reference value of the resistivity index (RI) obtained in renal inter-lobar arteries of both kidneys in the studied cats with no correlation between the renal resistivity index and the animal sex or body weight.

## Data Availability

The data that support the findings of this study are available from the corresponding author upon reasonable request.
